# Supplementary material for: Racial/ethnic disparities in exposure to COVID-19, susceptibility to COVID-19 and access to health care – findings from a U.S. national cohort
Source: medRxiv. 2022 Jan 12:2022.01.11.22269101. Preprint. [Version 1] doi: 10.1101/2022.01.11.22269101 (PMC8764735; doi:10.1101/2022.01.11.22269101)
Supplement: 1 [file NIHPP2022.01.11.22269101V1-supplement-1.pdf]

medRxiv preprint doi: https://doi.org/10.1101/2022.01.11.22269101; this version posted January 12, 2022. The copyright holder for this preprint (which was not certified by peer review) is the author/funder, who has granted medRxiv a license to display the preprint in perpetuity.
It is made available under a CC-BY 4.0 International license .

Supplemental Table 1. Demographic and Socioeconomic Characteristics of the Chasing COVID Cohort (C3) Participants who were Seronegative and Re-Tested - United States, Enrolled from March 28 - April 20, 2020 (N = 3,422)

	Total	Hispanic	Black NH	Asian\Pacific Islander NH	White NH	Other NH	P-value
**Total, n (%*)**	3,422 (100.00)	500 (14.61)	279 (8.15)	219 (6.4)	2,312 (67.56)	112 (3.28)	
**Age**							< 0.001
Mean (SD)	44 (15.16)	39.51 (13.13)	40.5 (12.90)	33 (13.18)	46.62 (15.43)	44.33 (13.57)	

Median (IQR)	42 (32, 56)	37 (30, 48)	37 (30, 51)	33 (26, 41)	45 (34, 60)	43 (35, 53)	
**Age Category, n (%)**							< 0.001
18-29	617 (18.03)	120 (24.00)	57 (20.43)	84 (38.36)	342 (14.79)	14 (12.50)	
30-39	934 (27.29)	166 (33.20)	100 (35.84)	76 (34.70)	566 (24.48)	26 (23.21)	
40-49	654 (19.11)	104 (20.80)	50 (17.92)	30 (13.70)	430 (18.60)	40 (35.71)	
50-59	514 (15.02)	62 (12.40)	40 (14.34)	11 (5.02)	388 (16.78)	13 (11.61)	
60+	703 (20.54)	48 (9.60)	32 (11.47)	18 (8.22)	586 (25.35)	19 (16.96)	
**Gender, n (%)**							< 0.001
Male	1,516 (44.30)	219 (43.80)	124 (44.44)	88 (40.18)	1,033 (44.68)	52 (46.43)	
Female	1,810 (52.89)	267 (53.40)	147 (52.69)	128 (58.45)	1,221 (52.81)	47 (41.96)	
Non-Binary	96 (2.81)	14 (2.80)	8 (2.87)	3 (1.37)	58 (2.51)	13 (11.61)	
**Education, n (%)**							< 0.001
Less than 12th grade	37 (1.08)	10 (2.00)	2 (0.72)	2 (0.91)	22 (0.95)	1 (0.89)	
12th grade/GED	278 (8.12)	71 (14.20)	33 (11.83)	6 (2.74)	155 (6.70)	13 (11.61)	
Some college (1-3 years)	815 (23.82)	151 (30.20)	112 (40.14)	38 (17.35)	488 (21.11)	26 (23.21)	
College (4 or more years)	2,292 (66.98)	268 (53.60)	132 (47.31)	173 (79.00)	1,647 (71.24)	72 (64.29)	
**Employment Status, n (%)**							< 0.001
Employed	2,102 (61.43)	284 (56.80)	181 (64.87)	131 (59.82)	1,436 (62.11)	70 (62.50)	
Out of Work	402 (11.75)	88 (17.60)	42 (15.05)	26 (11.87)	229 (9.90)	17 (15.18)	
Other	918 (26.83)	128 (25.60)	56 (20.07)	62 (28.31)	647 (27.98)	25 (22.32)	
**Income, n (%)**							< 0.001
Less than $35,000	870 (25.42)	174 (34.80)	113 (40.50)	47 (21.46)	494 (21.37)	42 (37.50)	
$35,000 - $49,999	383 (11.19)	77 (15.40)	43 (15.41)	16 (7.31)	231 (9.99)	16 (14.29)	
$50,000 - $69,999	505 (14.76)	75 (15.00)	56 (20.07)	30 (13.70)	333 (14.40)	11 (9.82)	
$70,000 - $99,999	592 (17.30)	73 (14.60)	30 (10.75)	43 (19.63)	436 (18.86)	10 (8.93)	
$100,000+	993 (29.02)	90 (18.00)	33 (11.83)	72 (32.88)	768 (33.22)	30 (26.79)	
Don't know	79 (2.31)	11 (2.20)	4 (1.43)	11 (5.02)	50 (2.16)	3 (2.68)	
**Any Children <18, n (%)**							< 0.001
No	2,596 (75.86)	326 (65.20)	183 (65.59)	154 (70.32)	1,853 (80.15)	80 (71.43)	
Yes	826 (24.14)	174 (34.80)	96 (34.41)	65 (29.68)	459 (19.85)	32 (28.57)	

IQR: Interquartile Range, NH: non-Hispanic, n: number, SD: standard deviation, US: United States, and %: percentage

*P-value based on the kruskall wallis test for means and Chi Square for frequencies

**Supplemental Table 2. Measures of Exposure, Susceptibility and Access to Care – Among Participants who were Seronegative and Re-Tested (N = 3,422)**							
	**Overall (N = 6,740)**	**Hispanic (N=1,308)**	**Black NH (N=899)**	**Asian\Pacific Islander (N=465)**	**White NH (N=3,846)**	**Other (N=222)**	**P-value**

medRxiv preprint doi: https://doi.org/10.1101/2022.01.11.22269101; this version posted January 12, 2022. The copyright holder for this preprint (which was not certified by peer review) is the author/funder, who has granted medRxiv a license to display the preprint in perpetuity. It is made available under a CC-BY 4.0 International license .

**Measures of Exposure: Inability to Impose Social Distance**							
**Structural measures**							
Living in an urban area	1,430 (41.79)	219 (43.80)	140 (50.18)	116 (52.97)	906 (39.19)	49 (43.75)	<0.001
Living in an multidwelling building	1,348 (39.39)	206 (41.20)	144 (51.61)	93 (42.47)	859 (37.15)	46 (41.07)	<0.001
Ability to avoid public transportation	218 (6.37)	38 (7.60)	37 (13.26)	7 (3.20)	128 (5.54)	8 (7.14)	<0.001
Summative index (structural measures)							
Median (IQR)	1 (0,2)	1 (0,2)	1 (0,2)	1 (0,2)	1 (0,1)	1 (0,2)	<0.001
**Work related measures**							
Not able to work from home	849 (24.81)	134 (26.80)	87 (31.18)	42 (19.18)	554 (23.96)	32 (28.57)	0.01
Will not get paid if at home	632 (18.47)	104 (20.80)	70 (25.09)	38 (17.35)	398 (17.21)	22 (19.64)	0.01
Does not have sick leave	746 (21.80)	116 (23.20)	82 (29.39)	46 (21.00)	474 (20.50)	28 (25.00)	0.01
Could lose job or business if unable to go to work	597 (17.45)	113 (22.60)	69 (24.73)	38 (17.35)	353 (15.27)	24 (21.43)	<0.001
Job can only be done in workplace	911 (26.62)	157 (31.40)	98 (35.13)	50 (22.83)	577 (24.96)	29 (25.89)	<0.01
Essential worker	282 (8.24)	34 (6.80)	32 (11.47)	15 (6.85)	189 (8.17)	12 (10.71)	0.15
Summative index (work related measures)							<0.001
Median (IQR)	1 (0,2)	1 (0,3)	2 (0,3)	1 (0,2)	0 (0,2)	1 (0,3)	
Summative index: structural and work-related measures							
Median (IQR)	2 (1,3)	2 (1,4)	3 (1,4)	2 (1,3)	2 (1,3)	2 (1,4)	<0.001
More exposure risk: index >2	1,115 (32.58)	189 (37.80)	130 (46.59)	69 (31.51)	687 (29.71)	40 (35.71)	<0.001
**Measures of Susceptibility**							
Age 60+	703 (20.54)	48 (9.60)	32 (11.47)	18 (8.22)	586 (25.35)	19 (16.96)	<0.001
Chronic Lung Disease	103 (3.01)	17 (3.40)	9 (3.23)	5 (2.28)	64 (2.77)	8 (7.14)	0.10
Asthma (current)	389 (11.37)	62 (12.40)	35 (12.54)	10 (4.57)	261 (11.29)	21 (18.75)	<0.01
T2 Diabetes	231 (6.75)	44 (8.80)	29 (10.39)	5 (2.28)	140 (6.06)	13 (11.61)	<0.01
Serious heart condition	865 (25.28)	115 (23.00)	89 (31.90)	20 (9.13)	613 (26.51)	28 (25.00)	<0.001
Kidney disease	50 (1.46)	7 (1.40)	2 (0.72)	1 (0.46)	39 (1.69)	1 (0.89)	0.45
Immunocompromised	108 (3.16)	13 (2.60)	7 (2.51)	3 (1.37)	80 (3.46)	5 (4.46)	0.34
HIV	161 (4.70)	25 (5.00)	30 (10.75)	3 (1.37)	97 (4.20)	6 (5.36)	<0.001
Daily smoker	343 (10.02)	58 (11.60)	49 (17.56)	8 (3.65)	202 (8.74)	26 (23.21)	<0.001
Summative index (measures of susceptibility)							
Median (IQR)	1 (0, 1)	0 (0,1)	1 (0,1)	0 (0,1)	1 (0,1)	1 (0,2)	<0.001
More susceptible: index >1	795 (23.23)	98 (19.60)	78 (27.96)	15 (6.85)	570 (24.65)	34 (30.36)	<0.001
**Healthcare Access**							
Does not have one person as doctor	808 (23.61)	145 (29.00)	74 (26.52)	69 (31.51)	483 (20.89)	37 (33.04)	<0.001

medRxiv preprint doi: https://doi.org/10.1101/2022.01.11.22269101; this version posted January 12, 2022. The copyright holder for this preprint (which was not certified by peer review) is the author/funder, who has granted medRxiv a license to display the preprint in perpetuity.
It is made available under a CC-BY 4.0 International license .

Did not see doctor due to cost	510 (14.90)	94 (18.80)	58 (20.79)	41 (18.72)	295 (12.76)	22 (19.64)	<0.001
Did not see doctor due to immigration	27 (0.79)	13 (2.60)	4 (1.43)	5 (2.28)	2 (0.09)	3 (2.68)	<0.001
No insurance	384 (11.22)	88 (17.60)	56 (20.07)	27 (12.33)	195 (8.43)	18 (16.07)	<0.001
Summative index (healthcare access)							
Median (IQR)	0 (0,1)	1 (0,2)	1 (0,2)	0 (0,1)	0 (0,1)	1 (0,1)	<0.001
More difficulty with access: index >0	1,213 (35.45)	217 (43.40)	121 (43.37)	97 (44.29)	726 (31.40)	52 (46.43)	<0.001
IQR: interquartile range, SD: standard deviation							
P-value statistic is based on the chi-square test for categorical data or the Kruskal Wallis for the summative indices							

Supplemental Table 3. Proportion Hospitalized (Yes) or Seroconverted (Yes) By Exposure Level Within Race/Ethnicity Strata

	**Overall (N = 3,422)**	**White NH (N=2,312)**	**Non-White (N=1,110)**	**Non-White (N=1,110)**				**Chi-Square P-Value for Differences by Race/Ethnicity**
				**Hispanic (N=500)**	**Black NH (N=279)**	**API NH (N=219)**	**Other NH (N=112)**	
**Seroconversion - N(%)**	161 (4.70)	93 (4.02)	68 (6.13)	37 (7.40)	17 (6.09)	7 (3.20)	7 (6.25)	<0.01
Measure of Exposure								<0.001
Less exposure risk	86 (3.73)	53 (3.26)	33 (4.84)	20 (6.43)	6 (4.03)	5 (3.33)	2 (2.78)	
More exposure risk	75 (6.73)	40 (5.82)	35 (8.18)	17 (8.99)	11 (8.46)	2 (2.90)	5 (12.50)	
Susceptibility								0.03
Less susceptible	130 (4.95)	76 (4.36)	54 (6.10)	30 (7.46)	13 (6.47)	7 (3.43)	4 (5.13)	
More susceptible	31 (3.90)	17 (2.98)	14 (6.22)	7 (7.14)	4 (5.13)	0	3 (8.82)	
Healthcare Access								0.02
Less barriers to access	93 (4.21)	58 (3.66)	35 (5.62)	21 (7.42)	8 (5.06)	3 (2.46)	3 (5.00)	
More barriers to access	68 (5.61)	35 (4.82)	33 (6.78)	16 (7.37)	9 (7.44)	4 (4.12)	4 (7.69)	
	**Overall (N = 6740)**	**White NH (N=1403)**	**Non-White (N=3,846)**	**Non-White (N=3,846)**				
				**Hispanic (N=1308)**	**Black NH (N=899)**	**API NH (N=465)**	**Other NH (N=222)**	
**Hospitalization**	401 (5.95)	185 (4.81)	216 (7.46)	108 (8.26)	78 (8.68)	13 (2.80)	17 (7.66)	<0.001
Measure of Exposure								<0.001
Less exposure risk	178 (4.30)	99 (3.85)	79 (5.03)	35 (4.95)	28 (6.41)	9 (3.01)	7 (5.51)	
More exposure risk	223 (8.59)	86 (6.76)	137 (10.35)	73 (12.15)	50 (10.82)	4 (2.41)	10 (10.53)	
Susceptibility								<0.001
Less susceptible	258 (4.88)	119 (4.07)	139 (5.88)	65 (6.07)	53 (7.60)	11 (2.53)	10 (6.13)	

medRxiv preprint doi: https://doi.org/10.1101/2022.01.11.22269101; this version posted January 12, 2022. The copyright holder for this preprint (which was not certified by peer review) is the author/funder, who has granted medRxiv a license to display the preprint in perpetuity.
It is made available under a CC-BY 4.0 International license .

More susceptible	143 (9.84)	66 (7.14)	77 (14.56)	43 (18.07)	25 (12.38)	2 (6.67)	7 (11.86)	
Healthcare Access								<0.001
Less barriers to access	130 (3.52)	78 (3.23)	52 (4.08)	26 (4.65)	18 (4.63)	4 (1.71)	4 (4.35)	
More barriers to access	271 (8.89)	107 (7.48)	164 (10.12)	82 (10.95)	60 (11.76)	9 (3.90)	13 (10.00)	
*Chi-sq assessed differences in proportion by White NH and Non-White								

**Supplemental Table 4. Modification of the association between race/ethnicity and seroconversion by (A) inability to impose social distance, (B) susceptibility and (C) healthcare access – N = 3,422**					
					**AOR (95% CI) for race/ethnicity effect within exposure strata**
Measure of Exposure	**White NH**		**Non-White**		**Non-White Versus White**
	**N Hospitalized /Denominator (%)**	**AOR (95% CI)**	**N Hospitalized /Denominator (%)**	**AOR (95% CI)**	
Less exposure risk	53/1625 (3.26)	1.00	33/682 (4.84)	**1.41 (0.89, 2.23)**	**1.41 (0.89, 2.23)**
More exposure risk	40/687 (5.82)	**1.66 (1.08, 2.56)**	35/428 (8.18)	**2.29 (1.42, 3.69)**	1.38 (0.85, 2.23)
AORs (95% CI) for less versus more within strata of race/ethnicity		**1.66 (1.08, 2.56)**		1.62 (0.98, 2.68)	
		**p=0.02**		p=0.06	
RERI (95% CI): measure of interaction on the additive scale				0.22 (-0.89, 1.32)	
				p=0.54	
Susceptibility	**White NH**		**Non-White**		**AOR (95% CI) for race/ethnicity effect within exposure strata**
	**N Hospitalized /Denominator (%)**	**AOR (95% CI)**	**N Hospitalized /Denominator (%)**	**AOR (95% CI)**	**Non-White Versus White**
Less susceptible	76/1742 (4.36)	1.00	54/885 (6.10)	1.36 (0.94, 1.96)	1.36 (0.94, 1.96)
More susceptible	17/570 (2.98)	0.62 (0.36, 1.07)	14/225 (6.22)	1.30 (0.71, 2.37)	**2.08 (1.00, 4.32)**
AORs (95% CI) for more versus less within strata of race/ethnicity		0.62 (0.36, 1.07)		0.96 (0.52, 1.76)	
		p=0.09		p=0.88	
RERI (95% CI): measure of interaction on the additive scale				0.31 (-0.55, 1.18)	
				p=0.47	
Healthcare Access	**White NH**		**Non-White**		**AOR (95% CI) for race/ethnicity effect within exposure strata**
	**N Hospitalized /Denominator (%)**	**AOR (95% CI)**	**N Hospitalized /Denominator (%)**	**AOR (95% CI)**	**Non-White Versus White**
Less barriers to access	58/1586 (3.66)	1.00	35/623 (5.62)	1.43 (0.92, 2.24)	1.43 (0.92, 2.24)
More barriers to access	35/726 (4.82)	1.23 (0.79, 1.92)	33/487 (6.78)	1.67 (1.03, 2.71)	1.36 (0.82, 2.23)
AORs (95% CI) for less versus more within strata of race/ethnicity		1.23 (0.79, 1.92)		1.16 (0.70, 1.93)	

medRxiv preprint doi: https://doi.org/10.1101/2022.01.11.22269101; this version posted January 12, 2022. The copyright holder for this preprint (which was not certified by peer review) is the author/funder, who has granted medRxiv a license to display the preprint in perpetuity.
It is made available under a CC-BY 4.0 International license .

		p=0.36		p=0.55	
RERI (95% CI): measure of interaction on the additive scale				0.01 (-0.91, 0.92)	
				p=0.99	

medRxiv preprint doi: https://doi.org/10.1101/2022.01.11.22269101; this version posted January 12, 2022. The copyright holder for this preprint (which was not certified by peer review) is the author/funder, who has granted medRxiv a license to display the preprint in perpetuity.
It is made available under a CC-BY 4.0 International license .
